# Toward a diagnostic CART model for Ischemic heart disease and idiopathic dilated cardiomyopathy based on heart rate total variability

**DOI:** 10.1007/s11517-022-02618-9

**Published:** 2022-07-09

**Authors:** Agostino Accardo, Luca Restivo, Miloš Ajčević, Aleksandar Miladinović, Katerina Iscra, Giulia Silveri, Marco Merlo, Gianfranco Sinagra

**Affiliations:** 1 Department Engineering and Architecture, University of Trieste, Trieste, Italy; 2 Cardiovascular Department, Azienda Sanitaria Universitaria Giuliano Isontina (ASUGI) and University of Trieste, Trieste, Italy; 3 Institute for Maternal and Child Health – IRCCS Burlo Garofolo, Trieste, Italy

**Keywords:** Computer-aided diagnosis, Heart rate variability, Ischemic heart disease, Dilated cardiomyopathy, Interpretable machine learning

## Abstract

Diagnosis of etiology in early-stage ischemic heart disease (IHD) and dilated cardiomyopathy (DCM) patients may be challenging. We aimed at investigating, by means of classification and regression tree (CART) modeling, the predictive power of heart rate variability (HRV) features together with clinical parameters to support the diagnosis in the early stage of IHD and DCM. The study included 263 IHD and 181 DCM patients, as well as 689 healthy subjects. A 24 h Holter monitoring was used and linear and non-linear HRV parameters were extracted considering both normal and ectopic beats (heart rate total variability signal). We used a CART algorithm to produce classification models based on HRV together with relevant clinical (age, sex, and left ventricular ejection fraction, LVEF) features. Among HRV parameters, MeanRR, SDNN, pNN50, LF, LF/HF, LFn, FD, Beta exp were selected by the CART algorithm and included in the produced models. The model based on pNN50, FD, sex, age, and LVEF features presented the highest accuracy (73.3%). The proposed approach based on HRV parameters, age, sex, and LVEF features highlighted the possibility to produce clinically interpretable models capable to differentiate IHD, DCM, and healthy subjects with accuracy which is clinically relevant in first steps of the IHD and DCM diagnostic process.

**Graphical abstract:**

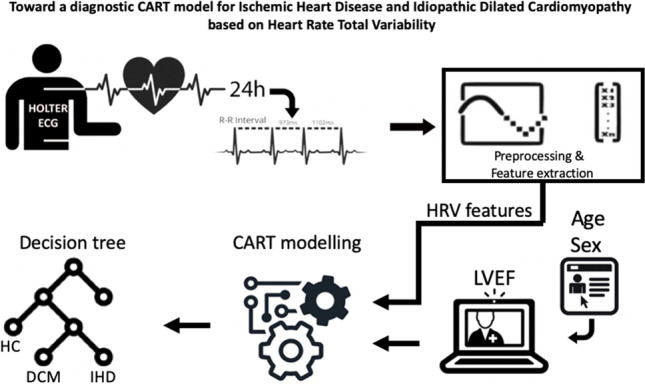

## Introduction

Ischemic heart disease (IHD), in its chronic stable form, is a subtle pathology due to its silent behavior before developing in unstable angina, myocardial infarction or, possibly, sudden cardiac death. This condition typically occurs when there is an imbalance between myocardial oxygen supply and demand, typically due to atherosclerotic heart disease. The diagnosis in the early stage of the IHD is necessary to improve clinical outcomes, which can often be challenging. Clinical diagnosis relies on the patient’s symptoms, especially chest pain, on the pathological ECG and on echocardiography, while only invasive coronary angiography, including the use of possibly toxic contrast means, can provide a definite diagnosis. At the same time, dilated cardiomyopathy (DCM) is a non-ischemic and non-valvular heart muscle disease frequently characterized by significant left ventricular (LV) or biventricular systolic dysfunction at the time of the diagnosis despite asymptomatic or scarcely symptomatic patients [1] reflecting a long period of asymptomatic silent disease progression [2]. Diagnosis of DCM, particularly in the early stages of the disease, can often be difficult and rely on advanced echocardiography (speckle tracking analysis), cardiac magnetic resonance imaging, including a comprehensive tissue characterization analysis, and genetic testing that often are not available or difficult to deliver to patients. Therefore, novel biomarkers, preferably based on non-invasive techniques, are needed.

Heart disease–related pathophysiologic changes and subsequent alteration of heart rate variability (HRV) can provide important prognostic information [3]. In addition, HRV-based biomarkers have a potentially important role in risk stratification for individuals with suspected heart disease [4]. Nevertheless, the diagnostic role of HRV differentiation between IHD and DCM is still in the early research stage [3]. Indeed, the features extracted from ECG alone may not be able to discriminate these pathological conditions, but they might be complementary to other clinical and instrumental parameters.

Despite the growing use of machine learning–based prediction models in medicine [5–9], clinicians still struggle to rely on these models in clinical practice [10]. Machine learning methods were also applied to produce heart disease detection and prediction models [11] based on clinical history and ECG features [12], magnetocardiography [13], photoplethysmography signal parameters [14], and HRV and blood pressure variability features [15].

One of the problems of the complex machine learning methods (e.g., random forest [16], neural networks [17]) is that the published results are mostly focused on a classification/regression model performance metric, but rarely on practical usability for prediction in medicine [18, 19]. Here is a need of ensuring that machine learning models used in healthcare are interpretable [10, 20]. The classification and regression tree (CART) [21] is an approach which produce interpretable models not only providing output information about a certain disease but also help to intrinsically evaluate the plausibility of the model by examining the selected thresholds and branches in comparison to the existing knowledge [22]. The classification tree modeling, even though in practice it might represent slightly lower accuracy in comparison to the black-box models [10], provides better interpretability and practical usability in clinical application. In addition, their simple visualization allows clinicians to follow a set of rules and thresholds for selected clinical and instrumental features.

The aim of this study was to investigate, by means of CART modeling, the predictive power of HRV features together with non-invasive clinical parameters to support the diagnosis in the early stage of ischemic heart disease and dilated cardiomyopathy.

## Methods

### Study population

In this study, we analyzed clinical data and processed ECG signals of 1133 subjects who were consecutively enrolled from December 2016 to October 2018 at the Cardiovascular Department of Trieste University Hospital (Trieste, Italy). In particular, the study encompassed 263 patients affected by IHD (207 males, aged 71 ± 10, and 56 females, age 76 ± 10 years), 181 patients suffering from DCM (111 males, age 59 ± 12 years, and 70 females, age 63 ± 15 years), and 689 healthy controls (321 males, age 62 ± 15 y, and 368 females, age 64 ± 16 years). The assessment of ischemic heart disease (IHD) was carried out from clinical and laboratory findings, and it was systematically confirmed by coronary angiography [23]. The IHD patients did not present acute coronary syndrome in the 3 months before the Holter monitoring. The DCM patients were enrolled after clinical assessment based on coronary artery disease sufficient to explain the dysfunction or in presence of a left ventricular ejection fraction (LVEF) < 50% without evidence of pressure or volume overload [1]. Coronary angiography was systematically performed in patients older than 35 years, with cardiovascular risk factors and/or without familial history for DCM*.* Patients with known trigger factors, such as toxic insults from alcohol or drugs, and tachyarrhythmias were also excluded. Both groups of patients were on beta-blocker pharmacological treatment. The exclusion criteria for healthy controls (HC) were the presence of peripheral artery disease, thyroid disorders, history of myocardial revascularization, hypertensive heart disease, pulmonary hypertension, or severe valvulopathy. The study was conducted according to the principles of the Declaration of Helsinki and was approved by the Trieste Hospital Ethic Committee (Project Number: N.O.43/2009, prot.2161). All participants released their written informed consent.

### Heart rate total variability acquisition and processing

All subjects underwent a 24 h Holter ECG recording using the ambulatory electrocardiographic recorder SpiderView (Sorin Group, Italy) with a sampling rate of 200 Hz. The RR intervals were extracted and labeled by using SyneScope analysis software (Sorin Group, Italy). The RR intervals were labeled as normal (N), premature ventricular contractions—ectopic beats (V), artifacts (A), and calibration (C). The RR interval records were cut into 5 min segments without overlap. Each RR 5-min segment was included in the analysis only if the longest ectopic beats subsequence (labeled with V) or the longest artifact subsequence (labeled with A) does not exceed 10 s. The RR marked with a calibration label was ignored. These segments were interpolated with cubic spline and resampled at 2 Hz, producing the heart rate total variability (HRV) signal. Subsequently, in each segment, linear and non-linear HRV parameters were extracted. In particular, the linear parameters MeanRR, SDNN, RMSSD, NN50, and pNN50 evaluating the RR variability were calculated directly from RR sequence [24], while in the frequency domain, the absolute powers in low (LF = 0.04–0.15 Hz) and high (HF = 0.15–0.40 Hz) frequency bands, related to the vagal and sympathetic nerve control on the heart rhythm, were estimated from the interpolated HRV signal. Moreover, the normalized low and high-frequency powers (LFn, HFn) and their ratio (LF/HF) were calculated from the latter parameters. The non-linear analysis was carried out by calculating Poincaré plot parameters (SD1, SD2, SD1/SD2), reflecting the short- and the long-term variability [25], estimating the fractal dimension (FD) [26] and the power-law beta exponent [27], quantifying the complexity of the system generating the signal. Finally, the median values of all parameters during 24 h were calculated and used as the input feature vector of the classifier.

### Classification method, features selection, and performance measurements

The CART method [21], used for diagnostic modeling because of its easy interpretability in the clinical domain, was employed to produce models capable of differentiating between three groups (IHD, DCM, and HC). The models were at first produced considering HRV features (Table 1) together with subjects’ age and sex. Three different models were considered: in the first set, all the 17 features were taken in consideration (Model_All_features_), in the second and third model, only the selected features were used as input of the CART. Stepwise regression algorithm, selecting only the most significant explanatory variables, and correlation analysis (excluding those variables presenting a regression coefficient less than 0.8) were used to operate the selection, producing Model_Stw_ and Model_Corr_, respectively. Stepwise regression, which was applied, is the step-by-step iterative algorithm for the selection of independent variables by adding or removing potential explanatory variables in succession and testing for statistical significance after each iteration.

**Table 1:** The set of linear and non-linear HRV features

HRV parameter	Definition
MeanRR (ms)	Mean of RR intervals
SDNN (ms)	Standard deviation of RR intervals
RMSSD (ms)	Root mean square of the squared differences of successive RR intervals
NN50	Number of differences of successive RR intervals greater than 50 ms
pNN50	Proportion of NN50 divided by the total number of RR intervals
LF (ms^2^)	Low-frequency power (from 0.04 to 0.15 Hz)
HF (ms^2^)	High-frequency power (from 0.15 to 0.40 Hz)
LF/HF	Low-frequency power/high-frequency power
LFn	Low-frequency power/total power
HFn	High-frequency power/total power
Beta exp (ms^2^/Hz)	Beta exponent
SD1 (ms)	Short-term variability of the RR sequence—from Poincarè Plot
SD2 (ms)	Long-term variability of the RR sequence—from Poincarè Plot
SD1/SD2	Short-term variability/long-term variability of the RR sequence
FD	Fractal dimension

Subsequently, another non-invasive parameter, the left ventricular volume ejection fraction (LVEF) obtained by the Simpson biplane method [28] useful to discriminate some heart pathologies, was also included. This parameter requires the execution of a further ecographic examination, which is performed in many cases and was added to understand if it was necessary to make it routine. Thus, further three different CART models were produced including all the features (Model_All_features+LVEF_) or, as previously, only those selected by stepwise (Model_Stw+LVEF_,) or low correlation (Model_Corr+LVEF_).

The CART uses an algorithm to construct the decision tree by essentially producing a set of rules represented by decisional nodes, branches, and leaves (i.e., terminal nodes) which are assigned to a class. The algorithm is based on a recursive segmentation (each non-leaf node has only two branches) and the generated decision tree is a simple structure in which each decision step can be divided into yes-or-no questions about each feature. The two steps of the CART are binary recursive partitioning to construct the complex binary tree and then prune it back to find an optimal subtree. In this work, the Gini coefficient, representing a variance estimate based on all comparisons of possible pairs of values in a subgroup, has been used as a loss function. Cross-validation was used as a technique to avoid overfitting and to produce a model that generalizes better to unseen data. The classification on the dataset was estimated using tenfold cross-validation. The process was then repeated 10 times, using each of the subsamples only once as the validation data. Therefore, the overall cross-validation accuracy was calculated as a mean of all 10 validation folds. To evaluate the trade-off between model interpretability and classification performance of produced decision tree, the obtained classification accuracy was compared with classification accuracy of other selected machine learning approaches: Logistic regression, Naïve Bayes, and support vector machine (SVM). All analyses were performed and implemented in MATLAB using the Statistics and Machine Learning toolbox.

## Results

The demographic and clinical characteristics of subjects included in the three considered groups, as well as, the mean values of linear and non-linear HRV parameters are reported in Table 2.

**Table 2:** Mean and standard deviation values of the features sets

	**IHD**	DCM	HC
Age	72 ± 11	61 ± 13	63 ± 15
Sex (M/F)	207/56	111/70	321/378
LVEF (%)	53 ± 13	44 ± 12	59 ± 6
HRV			
MeanRR (ms)	942 ± 145	880 ± 130	877 ± 138
SDNN (ms)	87 ± 68	85 ± 61	71 ± 53
RMSSD (ms)	71 ± 115	61 ± 111	38 ± 91
NN50	70 ± 90	65 ± 85	50 ± 73
pNN50	0.21 ± 0.27	0.19 ± 0.23	0.15 ± 0.21
LF (ms^2^)	350 ± 2400	521 ± 1500	460 ± 2200
HF (ms^2^)	640 ± 8100	626 ± 6005	276 ± 5800
LF/HF	0.97 ± 1.05	1.22 ± 1.30	2.06 ± 1.90
LFn	0.40 ± 0.20	0.44 ± 0.21	0.59 ± 0.22
HFn	0.60 ± 0.20	0.58 ± 0.21	0.44 ± 0.22
Beta exp (ms^2^/Hz)	0.67 ± 0.55	0.75 ± 0.58	1.06 ± 0.57
SD1 (ms)	46.4 ± 42.4	45.5 ± 40.9	34.8 ± 34.1
SD2 (ms)	95.5 ± 70.1	92.8 ± 57.3	83.1 ± 54.2
SD1/SD2	0.44 ± 0.15	0.44 ± 0.16	0.37 ± 0.14
FD	1.63 ± 0.15	1.63 ± 0.16	1.53 ± 0.16

When LVEF was not considered, the features selected by using the stepwise regression method were MeanRR, SDNN, LFn, FD, sex, and age (Model_Stw_), while the outcome of correlation analysis yielded in the selection of MeanRR, SDNN, LF, LF/HF, Beta exp, sex, and age (Model_Corr_). On the other hand, when LVEF was added to the other parameters, the features selected by using the stepwise regression method were MeanRR, pNN50, LF/HF, FD, sex, age, and LVEF (Model_Stw+LVEF_) and those identified by the correlation analysis were MeanRR, SDNN, LF, LF/HF, Beta exp, sex, age, and LVEF (Model_Corr+LVEF_). The selected features were used as input vector to produce six different decision tree models, as described in the methods section.

**Fig. 1:**
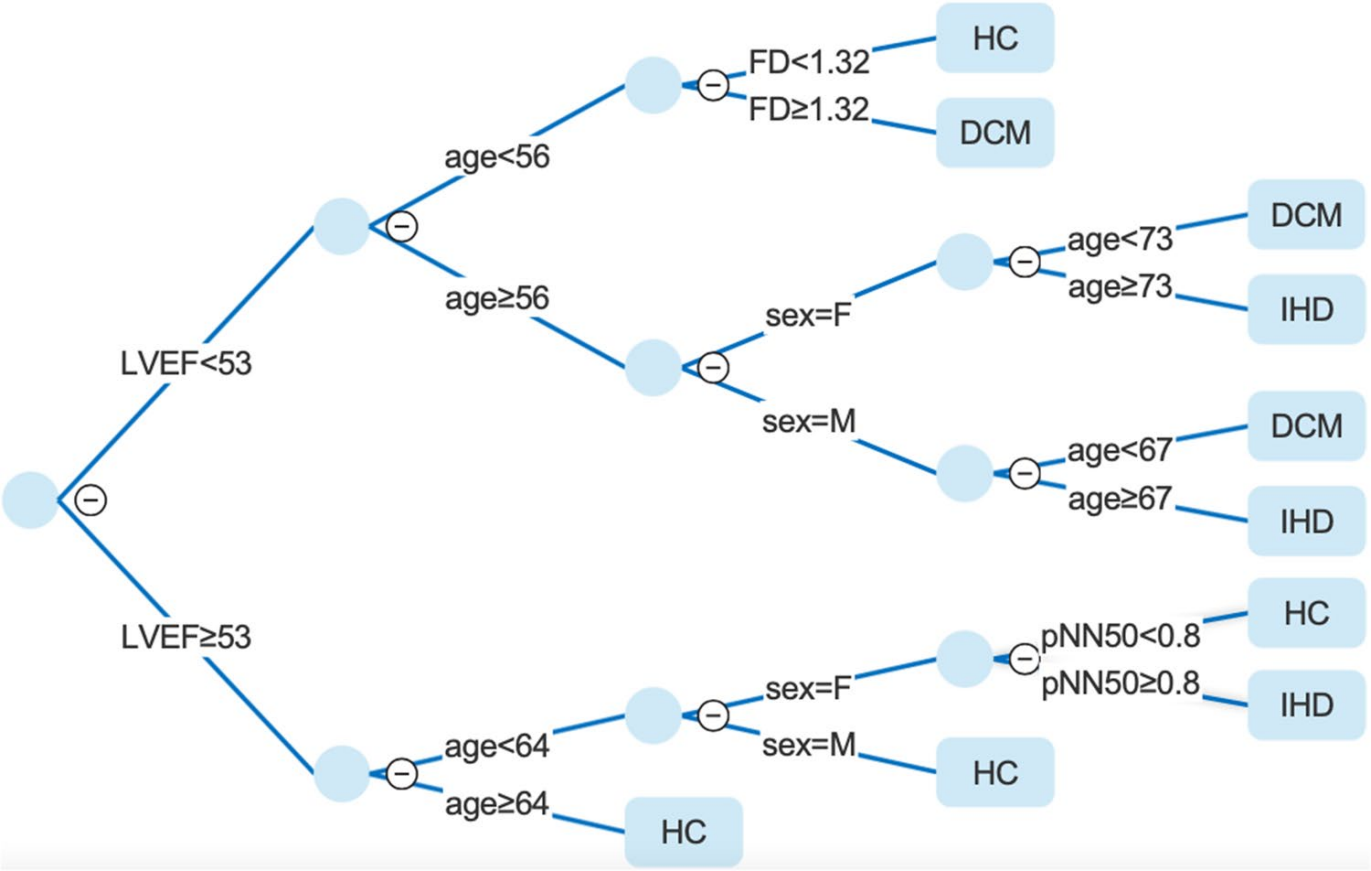
Decision tree model based on pNN50, FD, sex, age,and LVEF features. HC, healthy control; DCM, dilated cardiomyopathy; IHD, ischemic heart disease

**Fig. 2:**
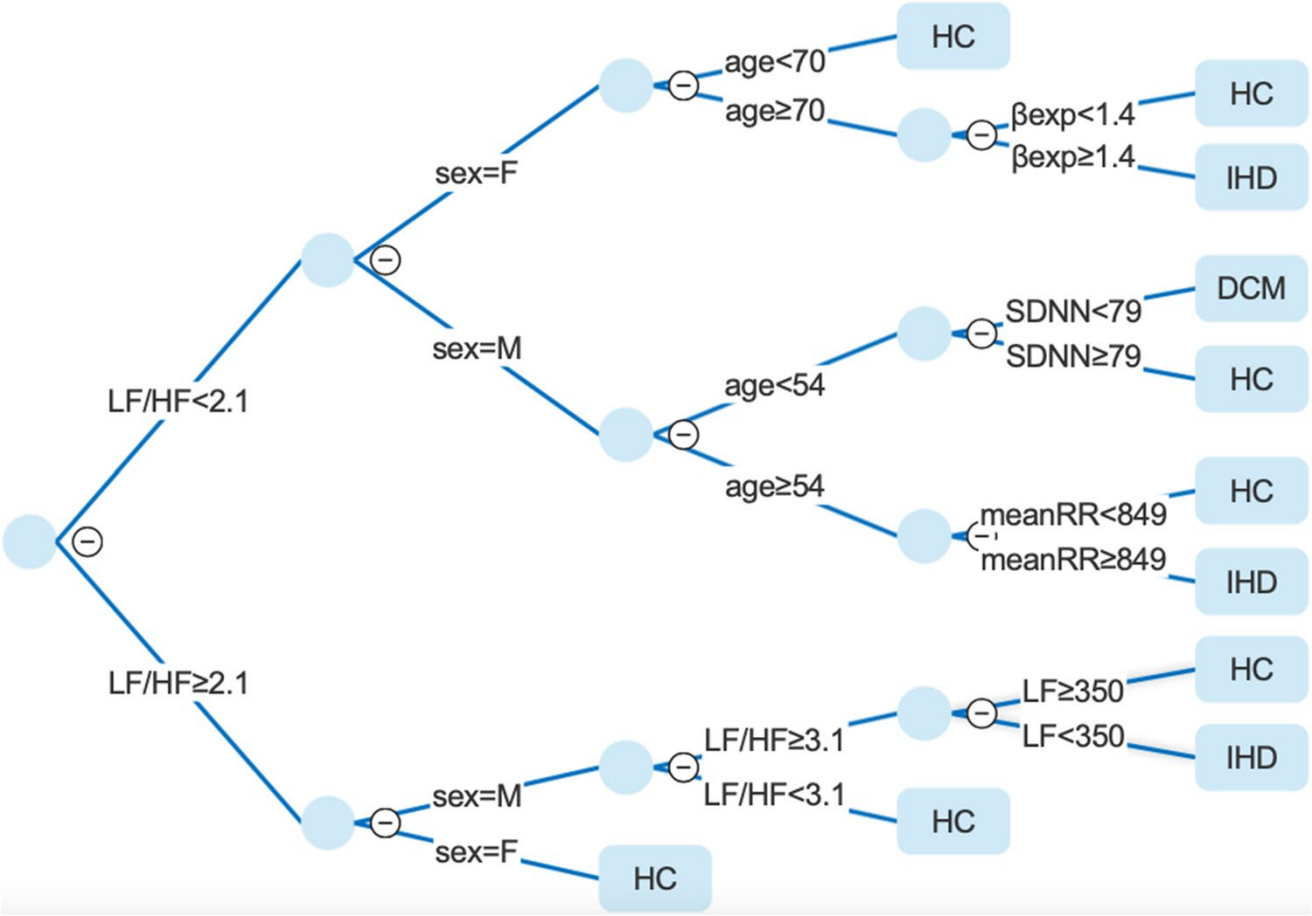
Decision tree model based on MeanRR, SDNN, LF, LF/HF, Beta exp, sex, age features. HC, healthy control; DCM, dilated cardiomyopathy; IHD, ischemic heart disease

Table 3 reports the features selected by CART approach (in bold) together with the performance metrics of produced models. In general, models which included LVEF showed a higher accuracy (about 10%) compared to those based only on HRV and demographic parameters. The highest accuracy on the test set was observed for the CART Model_Stw+LVEF_ (Fig. 1) while among the models based only on HRV and demographic parameters without LVEF the best classification performance was observed for the CART Model_Corr_ (Fig. 2). In addition, in Table 4 are reported AUC values for each group and model.

**Table 3:** Feature sets used as an input vector to produce the six models (in bold the features selected by CART algorithm) and their classification performance measures

	Model Features	CA	F1	Precision	Recall
Model_Stw_	**MeanRR, SDNN, LFn, FD, sex, age**	60.2%	58.6%	57.7%	60.1%
Model_Corr_	**MeanRR, SDNN, LF, LF/HF, Beta exp, sex, age**	61.4%	59.1%	58.1%	61.4%
Model_All_features_	MeanRR, SDNN, RMSSD, **NN50**, pNN50, **LF**, **HF**, LF/HF, LFn, **HFn**, Beta exp, SD1, SD2, SD1/SD2, FD, **sex**, **age**	60.3%	58.2%	57.2%	60.3%
Model_Stw+LVEF_	MeanRR, **pNN50**, LF/HF, **FD**, **sex**,**age**, **LVEF**	73.3%	71.3%	70.8%	72.9%
Model_Corr+LVEF_	MeanRR, SDNN, LF, LF/HF, Beta exp, **sex**, **age**, **LVEF**	72.8%	71.3%	70.8%	72.7%
Model_All_features+LVEF_	MeanRR, **SDNN**, RMSSD, NN50, pNN50, **LF**, HF, LF/HF, LFn, **HFn**, Beta exp, SD1, SD2, **SD1/SD2**, **FD**, **sex**, **age**, **LVEF**	72.6%	70.8%	70.3%	72.6%

**Table 4:** AUC values for each group and model

	AUC		
	HC	DCM	IHD
Model_Stw_	69.5%	62.7%	75.3%
Model_Corr_	70.8%	65.0%	73.8%
Model_All_features_	69.9%	63.0%	72.5%
Model_Stw+LVEF_	84.1%	83.0%	73.6%
Model_Corr+LVEF_	83.2%	83.5%	74.9%
Model_All_features+LVEF_	83.6%	84.3%	74.9%

The comparison of classification accuracies obtained by different machine learning approaches is reported in Table 5. The classification accuracies obtained with models produced with Logistic regression, Naïve Bayes, and support vector machine (SVM) methods were similar to those obtained with CART.

**Table 5:** Classification accuracies obtained by different machine learning algorithms

	CART	Logistic regression	Naive Bayes	SVM
Model_Stw_	60.2%	64.7%	63.3%	54%
Model_Stw_ + LVEF	73.3%	72.1%	74.2%	60.3%
Model_Corr_	61.4%	64%	61.6%	49.8%
Model_Corr_ + LVEF	72.8%	72.9%	71.7%	63.7%
Model_AllFeatures_	60.3%	65.4%	58%	50.4%
Model_AllFeatures_ + LVEF	72.6%	71.9%	62%	63.7%

## Discussion

Diagnosis of etiology in early-stage IHD and DCM patients may be challenging. IHD patients are generally asymptomatic or exhibit no typical signs and symptoms until the disease manifests as angina, myocardial infarction, or sudden cardiac death. Similarly, DCM represents a particular etiology of heart failure with reduced ejection fraction, frequently carrying a genetic background, which usually affects young patients with few co-morbidities, remaining asymptomatic for a long time. For this reason, there is research interest in the identification of novel, preferably non-invasive, biomarkers.

The main finding of this study is that the models including parameters extracted from the heart rate total variability signal are capable to differentiate three groups with accuracy, which is clinically relevant in first steps of the IHD and DCM diagnostic process. These findings support the hypothesis that HRV analysis emerges as an important, accessible, reproducible, supplementary tool for the IHD and DCM diagnosis.

Nowadays, different mathematical approaches for decision support systems have been proposed for the automatic classification of heartbeats and machine learning techniques have become a useful research diagnostic tool for physicians in the analysis of cardiovascular disease [29–32]. A recent study used artificial neural networks considering age, sex, and HRV features to classify ischemic heart disease patients and healthy subjects with an accuracy of 71.8% [29]. Moreover, considering also the left ventricular ejection fraction as a feature, even higher classification accuracy was obtained. Other authors identified only RR segments of DCM patients using HRV parameters as input of complex classifiers in which the CART was combined with other machine learning techniques [31, 32]. In particular, Thirugnaman et al. used different machine learning techniques applied to HRV parameters for identifying DCM and healthy ECG segments with an accuracy of 99.93% considering 22 ECG samples, 16 belonging to DCM and 6 to healthy subjects [32]. Moreover, Mahesh et al. used classification trees with logistic regression on a combination of linear and non-linear HRV parameters to identify ECG segments of DCM subjects with an accuracy of 95.61% (13 cardiomyopathy RR segments). Both studies took the data from a diagnostic ECG database [31]. Only Dua et al. used the classification and regression tree analysis to distinguish IHD patients from healthy subjects [30]. They analyzed the HRV signals of 20 subjects obtaining an accuracy of 81.1% applying principal component analysis to non-linear HRV parameters.

In our study, we aimed to discriminate not so much a specific pathologic or normal ECG segment belonging to subjects affected or not by cardiac pathology as rather to differentiate subjects belonging to the three groups (IHD, DCM, and HC) mainly by exploiting non-invasive HRV features. Among HRV parameters, MeanRR, SDNN, pNN50, LF, LF/HF, LFn, FD, and Beta exp were identified as the most informative. The produced interpretable decision tree models based only on HRV and demographic features, as well as including also LVEF, showed similar classification accuracies to those produced with logistic regression, Naïve Bayes, and support vector machine (SVM) methods. Even though in general the decision tree modeling might present a slightly lower accuracy in comparison to other commonly applied methods, we used CART algorithm because of its better interpretability and practical usability in clinical application. Models based only on HRV features and demographic parameters presented an accuracy of about 62%. From a clinical perspective, even the results obtained without LVEF (61.4%) is relevant, especially in the early differential diagnosis phase, as it allows to avoid invasive coronary angiography in selected patients. In the CART model without LVEF Model_Corr_, the most important feature is the LF/HF, which reflects sympatho-vagal balance that can be altered in the patients affected by cardiomyopathies [33]. In fact, it can be observed a denser grouping of the pathologies, and exclusive existence of DCM, in the upper subtree. We also observed that in the bottom subtree the final decision of IHD classification is based again on LF/HF and LF parameters, which confirm their high discriminatory power. The second most important feature observed in the CART Model_Corr_ is sex, which in the upper subtree is strictly related to patient age. It has been already reported that DCM affects men more commonly than women [34], which is also observable in our CART Model_Corr_. Regarding the age, the identified threshold of 54 years for men, is in line with previous findings, as most of DCM male patients become symptomatic between 20 and 60 years [35]. Finally, the SDNN is an additional parameter that allows fine classification between DCM and HC, as it reflects all the long-term HRV components and it is sensitive to low frequencies heart rate alteration present in DCM. Regarding IHD, in the CART Model_Corr_ was observed that it is more likely to be affected by the disease if the subject is older independently of sex. Furthermore, for beta exponent, a non-linear parameter related to the complexity of the signal generators is more likely to be altered in the patients affected with IHD [36], which can be also observed in produced decision tree.

The inclusion of LVEF parameter beside the HRV ones made it possible to improve the performance by about 10%. In particular, we observed the highest accuracy (73.3%) for the model based on pNN50, FD, sex, age, and LVEF features (Fig. 1). Concerning CART Model_Stw+LVEF_, the most important feature is LVEF. Our tree confirms that the cut-off for the diagnosis of DCM is around 50%. Indeed, in the subtree where the LVEF is higher than 53%, there are no branches to DCM identification. Moreover, we also observed the high relation of LVEF with patient age. In particular, the CART Model_Stw+LVEF_ showed that if the subject has high LVEF and it is older than 64 years old, it is more probable that it belongs to HC group. In the cases of LVEF below the 54% cut-off, the sex differences plays important role. Indeed, in our model it can be observed that females and males have different age thresholds to be classified as DCM or IHD (male age < 67, female age < 73).

LVEF showed that the model mainly based on HRV parameters classifies better IHD subjects than DCM and vice versa the model which takes into account also LVEF classifies better DCM subjects than IHD. This fact clearly indicates the contribution of the LVEF parameter, as discriminatory feature to identify the DCM but confounding to discriminate IHD patients. In order to further improve the classification performance, future studies could also take into account the circadian rhythm related physiological parameters variation [37–39].

## Conclusions

In conclusion, the proposed approach based on HRV parameters, age, sex, and LVEF features highlighted the possibility to produce clinically interpretable models capable to differentiate IHD, DCM, and healthy subjects with accuracy which is clinically relevant in first steps of the IHD and DCM diagnostic process. These results support the hypothesis that HRV analysis emerges as an important, accessible, reproducible, complementary tool for the IHD and DCM diagnosis, potentially avoiding invasive and toxic exams especially in healthy subject cases.
